# Distribution and retention trends of physician-scientists in Japan: a longitudinal study

**DOI:** 10.1186/s12909-019-1840-3

**Published:** 2019-10-28

**Authors:** Masatoshi Ishikawa

**Affiliations:** 1 Takemi Program in International Health, Harvard T.H. Chang School of Public Health, Boston, Massachusetts USA; 2 Health Services Research & Development Center, University of Tsukuba, Tsukuba, Ibaraki Japan

**Keywords:** Physician-scientists, Retention rate, Longitudinal study, Japan

## Abstract

**Background:**

Physician Scientists (PSs) play a significant role in medical science because of their clinical practice and research expertise. Although it is important to analyze the distribution and retention trends in the number of PSs in Japan, research on this topic has been insufficient. Thus, the purpose of this study is to analyze PSs distribution and retention trends, identify factors related to their retention, and consider the policy implications.

**Method:**

I analyzed individual data from 1996 to 2016 from a national census survey that had been administered by the national government of Japan every 2 years. The number of PSs in 1996 and 2016 were 4930 (2.1% of all physicians) and 5212 (1.6%), respectively. I conducted a descriptive analysis and identified retention trends. I then used a multivariable logistic regression analysis to identify the factors related to the retention of PSs.

**Results:**

Between 1996 and 2016, the total number of PSs in Japan increased by 6%. The number of PSs aged 39 years or younger decreased by 48%, while those aged between 55 and 69 increased by 91%, indicating a notable decrease in the number of PSs under the age of 39. From 2014 to 2016, the annual retention rate of PSs was estimated to be 75.5%, which represented a low and stable rate compared to other physicians over the study period. The odds of continuing to practice as a PS were significantly higher for those who have between 15 to 29 years of experience after qualification as a physician.

**Conclusion:**

This study indicates that it is likely for the total number of PSs to decrease in the future. Although the Japanese government has implemented various measures to retain PSs, these have not been effective. Possible new interventions to address this problem include increasing the knowledge of medical students and younger physicians of the role of PSs and the benefits of a career as a PS, providing specific career paths for PSs, securing specific positions for PSs, and increasing the compensation for PSs.

## Background

Physician-scientists (PSs) are physicians with a medical degree who spend more time on research than on clinical practice compared to practicing physicians. The term PS was coined in the early 1900s by Samuel Meltzer [1]. In the U.S., the data has suggested that PSs will soon “go extinct”, and as the number of biomedical researchers has increased, the number of PSs has continued to decrease [2–4]. Young physicians tend to avoid careers in medical science because it usually involves uncertainty from the perspective of their career path and salaries [5, 6]. According to a 2014 report issued by the National Institute of Health (NIH), in the U.S., there were 14,000 PSs (making up 1.5% of the physicians in the country), and since then, this figure has remained low compared to other physicians [7]. While PSs may be a minority compared to other physicians and scientists, they play an important role in closing the gaps between clinical practice and medical research, because of their clinical and research expertise [7].

In Japan, there has also been an increasing awareness and interest in the shortage of PSs. In 2009, the Association of Japanese Medical Colleges issued a statement that expressed concern about the future of medical research in Japan and pointed out the urgent necessity for the government to address the situation [8]; and in 2010, four academic societies for basic medicine issued a statement expressing a similar concern [9]. This statement argued that the number of younger physicians who pursue a career as a PS was decreasing because the government had cut research grant funding due to financial difficulties, and in 2004, the government introduced mandatory two-year postgraduate training for all medical graduates. As a consequence of these policies, the proportion of medical research publications in Japan decreased, suggesting that in terms of medical research, Japan was lagging behind other countries and this was an important policy issue to be addressed [10].

In the U.S., U.K., and Canada, to solve the shortage of PSs, several universities provide MD-PhD programs [11–13]. In addition, with support from the National Institutes of Health (NIH), some U.S. universities operate medical scientist training programs, in order to nurture PSs [11]. Students enrolled in these programs are exempt from tuition fees and receive stipends from the NIH for living costs and research expenses. Similarly, 2012 onward in Japan, with the funding of the Minister of Education, Culture, Sports, Science and Technology (MEXT), the number of MD-PhD courses and PS training courses increased in medical schools [14].

The changes in the number of PSs in Japan are captured in the Survey of Physicians, Dentists, and Pharmacists, which is a biennial survey conducted by the Japanese Ministry of Health, Labour and Welfare (MHLW) and the response rate is estimated to be approximately 90% [15]. However, detailed statistics and individual data, such as changes in the number of physicians by age group and their career paths, are not disclosed [16]. Koike et al. obtained detailed data with permission from the MHLW and studied the trends in PSs by analyzing the data. They reported that while the number of younger PSs decreased from 1996 to 2008, the total number remained almost unchanged [17]. However, their study was published in 2010 and they did not examine data from 2008, or the measures implemented to nurture or secure PSs such as MD-PhD courses and PS training courses [14].

Using the individual data from the Survey of Physicians, Dentists, and Pharmacists conducted by MHLW, it is possible to investigate empirically what types of physicians decide to become PSs. This research would help to generate data that could be used to examine the reasons behind the uneven distribution of PSs from the perspectives of geography, gender, and age. In addition, although it is important to analyze the distribution and retention trends of PSs and the factors that relate to their retention, there has been insufficient research in this area.

Hence, the objectives of the present study are to evaluate the recent retention trends of PSs in Japan, identify the factors associated with their retention, propose policy implications, and suggest effective intervention methods.

## Method

I obtained approval from the MHLW to use the individual data from this survey for this research. In this study, for each physician in 1996, 2006, and 2016, data on the following were evaluated: registration number, gender, age, years of experience, workplace or facility type (municipality and medical institution type), and area of practice. Physicians who graduated in 2004 advanced to mandatory two-year postgraduate training and became clinicians or physician scientists in 2006. Therefore, since 2004 was a transition period, 2006 was set as a breakpoint. The PSs were identified by the type of facility indicated in their responses. I defined PSs as those who described themselves as (1) staff or graduate students at academic hospitals who were not involved in clinical practice or (2) staff at research institutions. As a result, the number of PSs who were identified in 1996 and 2016 were 4930 (2.1% of all physicians) and 5212 (1.6%), respectively.

To differentiate the PSs geographically, 344 Secondary Medical Areas (SMAs) were identified and used for this study. The municipality borders that altered because of mergers were adjusted based on the borders in 2016. Regarding geography, there are no rural criteria in Japan comparable to the standards of the U.S. Office of Management and Budget [18]; thus, the classification used were the MHLW classification position statements regarding the demand for physicians [19]. The SMAs were then classified into three categories based on the combination of population size and population density in 2016: the first group (urban), second group (intermediate), and third group (rural). Based on the classification used by MHLW, the first group (urban) consisted of areas with a population of at least 1 million or a population density of at least 2000 people/km^2^. The second group (intermediate) consisted of areas with a population of at least 100,000 or a population density of at least 200 people/km^2^. The third group (rural) consisted of areas that did not belong to the first or second groups. In this study, physicians who were in the same group of SMAs throughout the study period were considered retained physicians, and this applied to PSs.

The number of physicians per 100,000 people in each group of SMAs was calculated using the data for the total number of physicians and total population taken from the National Census [20]. To account for the differences between the years of the physician data (1996, 2006, and 2016) and the years of the population data (1995, 2005, and 2015), I applied the 1996 physician data to the 1995 population data, the 2006 physician data to the 2005 population data, and the 2016 physician data to the 2015 population data.

**Fig. 1:**
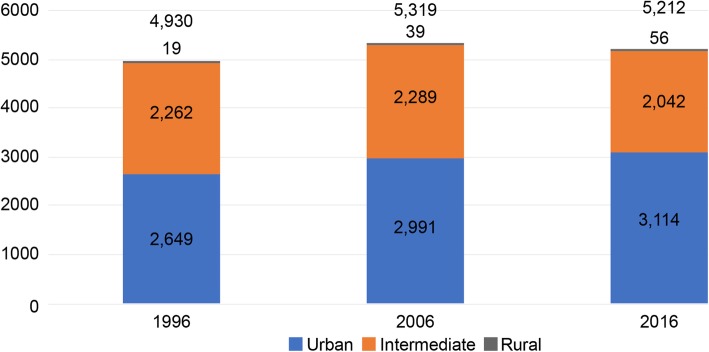
Distribution of PSs by geographic areas in 1996, 2006, and 2016

To analyze the date, first, I verified the distribution of PSs and the ratio of PSs per 100,000 residents by geographic area for 1996, 2006, and 2016 (Fig. 1). Next, I described the demographic and professional characteristics of the PSs in 1996, 2006, and 2016 (Table 1). I analyzed data from 2006 to compare the data before and after the introduction of the postgraduate mandatory training system. I then established a cohort dataset using the physician registration numbers. For the data from 1996 to 2016, I calculated the retention rate every 2 years and analyzed this trend. From this, the annual retention rates were obtained by calculating the square root of the biannual rates (Table 2).

**Table 1:** Demographic and professional characteristics of PSs in 1996, 2006, and 2016

	1996 Survey		2006 Survey		2016 Survey	
Total of subjects, n	4930		5319		5212	
% of all physicians	2.1%		1.9%		1.6%	
Sex, n, %						
Male	4441	90.1%	4591	86.3%	4280	82.1%
Female	489	9.9%	728	13.7%	932	17.9%
Age, n, %						
≦39	2207	44.8%	1671	31.4%	1168	22.4%
40–54	1616	32.8%	2352	44.2%	1958	37.6%
55–69	1005	20.4%	1114	20.9%	1917	36.8%
≧70	102	2.1%	182	3.4%	169	3.2%
Years of experience, n, %						
0–14	2215	44.9%	1682	31.6%	1187	22.8%
15–29	1701	34.5%	2358	44.3%	1917	36.8%
30–44	913	18.5%	1120	21.1%	1906	36.6%
≧45	101	2.0%	159	3.0%	202	3.9%
Qualification as a physician at age under or over 30 years, n, %						
< 30	3825	77.6%	4150	78.0%	4043	77.6%
≧30	1105	22.4%	1169	22.0%	1169	22.4%
Workplace, n, %						
Urban	2649	53.7%	2991	56.2%	3114	59.7%
Intermediate	2262	45.9%	2289	43.0%	2042	39.2%
Rural	19	0.4%	39	0.7%	56	1.1%

**Table 2:** Retention rate among PSs

	Period and number observed									
	1996–1998	1998–2000	2000–2002	2002–2004	2004–2006	2006–2008	2008–2010	2010–2012	2012–2014	2014–2016
Number of baseline, n	4930	5269	5393	5327	5255	5319	5223	5265	5075	4998
Still working as PSs, n (%)	3208	3270	3258	3157	3112	3221	3345	3324	3280	3243
Change in area of practice, n (%)	1118	1213	1289	1353	1511	1382	1296	1365	1185	1182
No report, n (%)	604	786	846	817	632	716	582	576	610	573
Estimated annual retention rate, %	75.6%	74.4%	73.8%	73.3%	73.3%	73.8%	75.2%	74.8%	75.4%	75.5%
Retention rate by year since registration as a physician %										
0–14	71.1%	70.6%	69.8%	69.3%	69.2%	69.3%	69.3%	69.2%	69.4%	69.3%
15–29	84.5%	82.9%	82.0%	81.6%	79.9%	80.4%	81.4%	80.1%	80.0%	81.8%
30–44	77.6%	74.4%	75.1%	76.6%	76.7%	75.9%	79.2%	78.1%	78.4%	78.0%
≧45	69.5%	69.2%	69.4%	69.3%	70.6%	69.6%	69.9%	69.7%	70.0%	69.9%

To identify the factors associated with retention in 2006 and 2016, a multivariable logistic regression analysis was conducted for respondents who identified themselves as PSs in 1996 and 2006 (Table 3). Continuation as a PS after 10 years was a dependent variable and gender, years of experience, qualified as a physician over 30 years of age (i.e., more than 5 years of experience predicted by graduation at age 25 after having entered medical college directly from high school), and workplace (geographic area) were independent variables.

**Table 3:** Logistic regression analysis of physicians in 1996 and 2006 who continue to work as PSs in 2006 and 2016

	OR	95% CI	*P*-value
1996–2006 cohort			
Sex			
Male		Reference	
Female	0.91	0.72–1.14	0.41
Years of experience			
0–14		Reference	
15–29**	3.48	3.00–4.02	< 0.01
30–44**	0.52	0.41–0.66	< 0.01
≧45	0.49	0.20–1.22	0.13
Qualified as a physician over 30 years old			
No		Reference	
Yes	0.76	0.53–0.68	< 0.01
Workplace			
Urban		Reference	
Intermediate	1.09	0.95–1.25	0.20
Rural	0.88	0.30–2.60	0.82
2006–2016 cohort			
Sex			
Male		Reference	
Female	0.99	0.82–1.20	0.91
Years of experience			
0–14		Reference	
15–29**	5.00	4.32–5.79	< 0.01
30–44	0.95	0.77–1.16	0.60
≧45*	0.38	0.17–0.86	0.02
Qualified as a physician over 30 years old			
No		Reference	
Yes	1.00	0.86–1.18	0.95
Workplace			
Urban		Reference	
Intermediate	1.09	0.96–1.25	0.19
Rural	1.46	0.60–3.55	0.40

* *p* < 0.05, ** *p* < 0.01

**Fig. 2:**
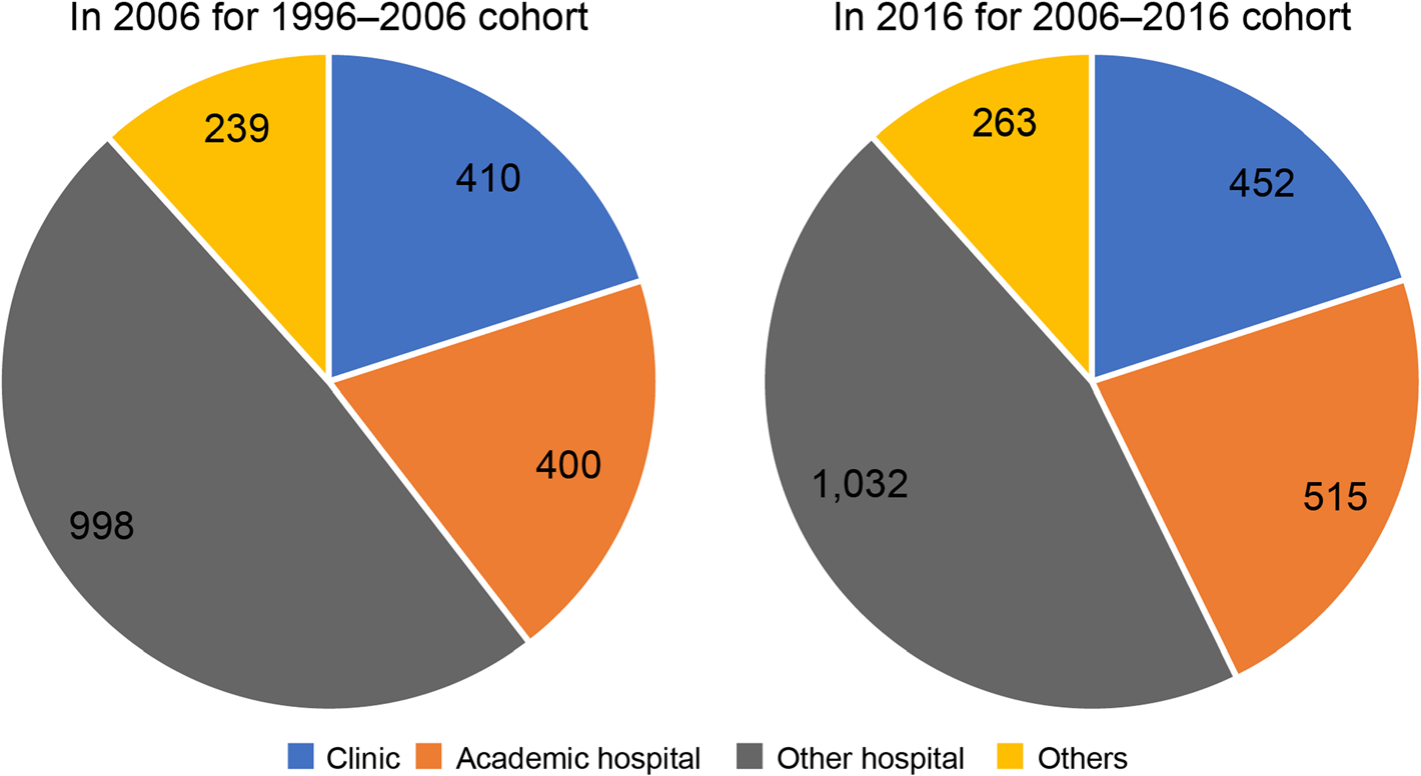
Types of institutions for those who left PSs in 2006–2016 and 1996–2006

**Fig. 3:**
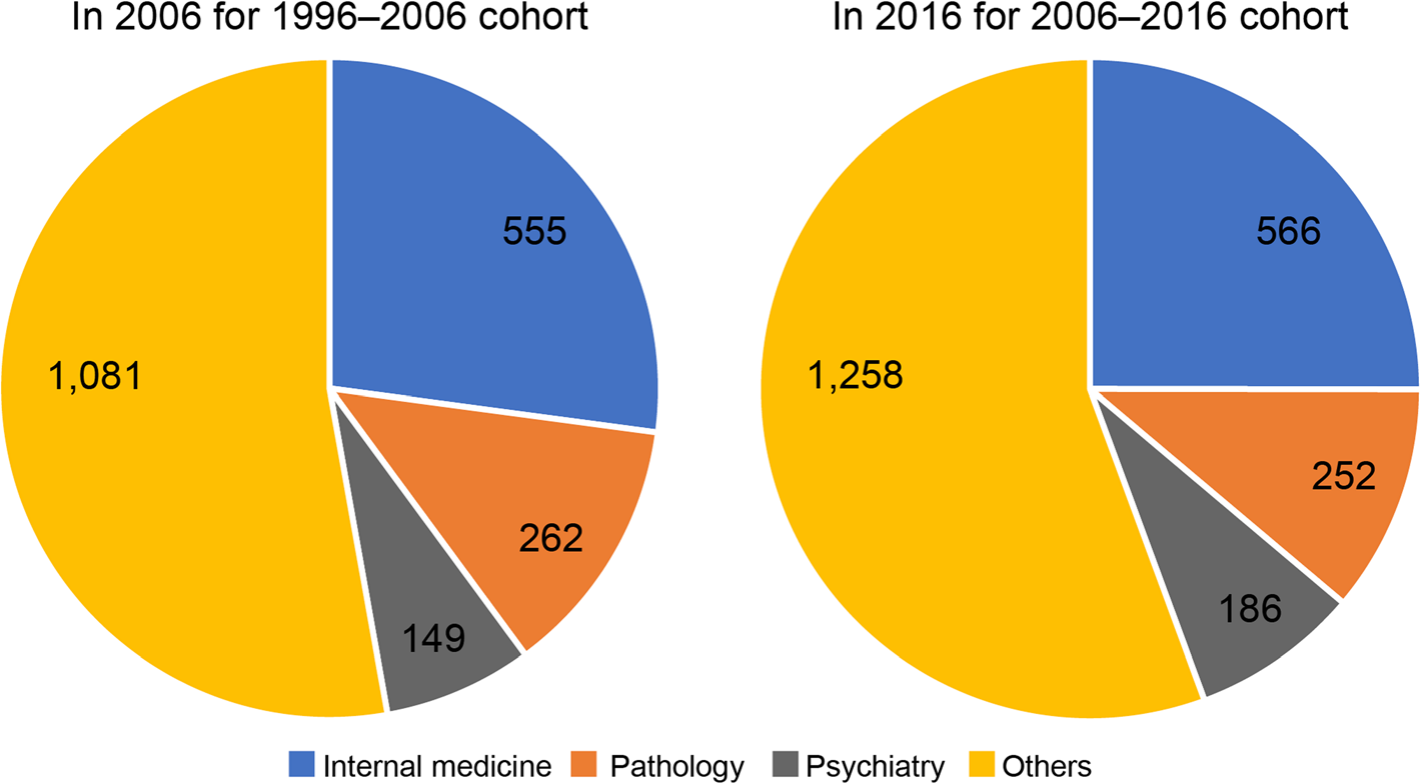
Specialties of those who left PSs in 2006–2016 for PSs in 1996–2006

In addition, I analyzed the types of institutions and specialties of the PSs who changed their career between 1996 and 2006 and between 2006 and 2016 (Figs. 2 and 3). Finally, I verified the types of specialty certificates held by PSs as of 2016 (Table 4).

**Table 4:** Types of specialty certificates held by PSs as of 2016

Total	5212	100.0%
No certificate	2664	51.1%
Internal medicine	403	7.7%
Pathology	345	6.6%
Pediatrics	270	5.2%
Psychiatry	193	3.7%
Others	1337	25.7%

For the statistical analyses, *P*-values of less than 0.05 were considered significant, and STATA 15.1 was used for all statistical analyses.

## Results

Figure 1 illustrates the distribution of PSs in Japan in 1996, 2006, and 2016. The number of PSs in 1996, 2006, and 2016 were 4930 (2.1% of all physicians), 5319 (1.9%), and 5212 (1.6%), respectively. The number of PSs increased from 4930 to 5212 by 6% during this period, although the percentage of physicians who were PSs decreased from 2.1 to 1.6%. Based on the rural criterion, between 1996 and 2016, the number of urban PSs increased by 18% whereas the number of rural PSs increased by 195%; thus, the urban versus rural differential decreased, although the number of rural PSs remained relatively small.

Table 1 presents the characteristics of PSs in 1996, 2006, and 2016. It should be noted that the number of PSs aged 39 years or younger decreased to 1168 (48%), between 1996 and 2016, while the number of PSs aged between 55 to 69 greatly increased to 1917 (91%) in the same period. Thus, the aging of PSs has advanced. The number of female PSs increased 1.9-fold between 1996 and 2016, and this led to the proportion of female PSs increasing from 9.9 to 17.9%.

The annual retention rates were calculated, and they are presented in Table 2. The proportion of PSs who remained in the same agency from 2014 to 2016 was 75.5%; this change is representative of general retention during the 1996–2016 period, which decreased from 75.6 to 75.5%.

Among younger PSs with 1–14 years of experience, the retention rates were relatively low, decreasing from 71.1% in 1996 to 69.3% in 2014. However, among older PSs with over 45 years of experience, the retention rates were also relatively low, increasing from 69.5% in 1996 to 69.9% in 2014. The ratios of those who did not report their PSs profession 2 years later (no-report ratio) ranged between 12.3% (1998) and 11.5% (2016), demonstrating no significant differences between the surveys.

Of the PSs in 1996, there was data available for 3827 in 2006, and 1780 still worked as PSs in 2006. Similarly, of the PSs in 2006, there was data available for 4285 in 2016, and 2023 still worked as PSs in 2016. The logistic regression analysis illustrated in Table 3 indicates that the odds of continuing to practice as a PS were significantly higher among females and among those who had been registered as a physician for 15 to 29 years (reference: 0 to 14 years, OR: 3.48, 95% CI: 3.00–4.02 for 1996–2006 cohort and OR: 3.48, 95% CI: 3.00–4.02 for 2006–2016 cohort). In the 1996–2006 cohort, the odds of continuing to practice as a PS were significantly lower among those with 30 to 44 years of experience (reference: 0 to 14 years, OR: 0.76, 95% CI: 0.53–0.68) and those who had qualified as physicians for over 30 years old (reference: qualified as physicians under 30 years old, OR: 0.52, 95% CI: 0.41–0.66). Additionally, in the 2006–2016 cohort, the odds of continuing to practice as a PS were significantly lower among those with 45 or more years of experience (reference: 0 to 14 years, OR: 0.38, 95% CI: 0.17–0.86). There was no significant relationship between the intention of continuing to practice as a PS and gender or workplace.

Figure 2 illustrates the details of the institutions of the PSs who changed their career between 1996 and 2006 and between 2006 and 2016. From this, it is evident that most of the PSs who changed their career worked for hospitals and clinics.

Figure 3 illustrates the details of the specialties of the PSs who changed their career between 1996 and 2006 and between 2006 and 2016. After the PSs changed their careers, internists were their most common new specialty, but pathology and psychiatry were also relatively popular fields.

Table 4 illustrates the specialty certificates of physicians working as PSs in 2016. A total of 2664, accounting for 51.1% of the 5212 PSs did not hold a specialist qualification. Most of the PSs qualified as internists, followed by pathologists, pediatricians, psychiatrists, and cardiologists. There were no data for specialists in 1996 and 2006 because the data collection was initiated in 2010.

## Discussion

### Result of statistics regarding the distribution and retention rates of physician scientists

From 1996 to 2016, the number of PSs in Japan slightly increased, and the number of younger PSs continued to decrease. This indicates that the trends up until 2008 demonstrated in the preceding study mentioned above have continued [16]. Also, from 2006, the number of female PSs and PSs aged 55 years or older have also increased. If this pattern continues, the number of PSs is likely to decrease further as more senior PSs resign because a large number of universities in Japan set a mandatory retirement age of 65 [21]. Therefore, this is an important political issue that is yet to be resolved.

The present study demonstrated that, in general, the annual retention rate among PSs was low compared to other physicians. For emergency medicine and rehabilitation, it has been demonstrated that physicians have low retention rates [15]. These low retention rates indicate that some PSs engage in short-term careers for research activities and then return to clinical practice.

Having 15 to 29 years of experience was identified as positively influencing a physicians’ choice to continue working as a PS. This may be because these PSs have accumulated a certain amount of career experience and are working in stable, tenured, and permanent positions, while the career path for younger PSs is uncertain [5, 6]. Meanwhile, no significant relationship was observed between the intention of continuing to practice as a PS and other variables such as gender and workplace.

### A decrease in the number of younger physician scientists

There are two issues in relation to the decrease in the number of younger PSs: first, few younger physicians become PSs; and second, the retention rate of younger PSs is low. The commencement of the mandatory, two-year residency training system in 2004 gave rise to the first issue, as younger physicians are now required to spend 2 years training prior to becoming PSs [9]. However, this is not a complete explanation because, after 2006, the number of younger physicians decreased. For younger physicians and medical students, early exposure to certain professions has a significant impact on their selected area of specialization [22] A possible reason might be that medical students who were originally considering pursuing a career as a PS changed their mind because they became interested in clinical medicine during their clinical training.

In addition, after the introduction of the mandatory residency training system in 2004, the proportion of physicians who underwent postgraduate training at a university hospital decreased from 72.5 to 44.7% between 2003 and 2004. Accompanying this, an increasing number of physicians are building careers without having a connection to a university [23], and university hospital departments currently have a weaker impact on physician careers. This has caused a decrease in the number of physicians enrolled in PhD courses and engaged in research [24]. Moreover, research budgets and the number of related positions have also reduced [9]. It is assumed that, as in the case of the U.S., younger physicians tend to avoid pursuing a career as a PS because of the uncertainty of this field [5, 6].

Since 2000, the retention rates of younger PSs has remained low, between 65 and 69%, and many physicians who left their PS job started working in hospitals. It has been pointed out that the shortage of teaching staff for basic medicine who have a clinical background affects medical education [24]. A large number of newly created medical schools in the 1970s [25] contributed to an increase in the number of basic medicine-related positions in medical schools at that time, and subsequently, this might be the reason for the increase in the number of PSs aged 55 years or older. In recent years, a large number of professors in basic medicine are PSs. However, the proportion of PSs in associate professor and similar positions has significantly decreased as few younger physicians engage in basic medicine.

While the number of female physicians has increased, the proportion of female PSs relative to the total number of PSs was 17.9% in 2016, which is lower than that of overall female physicians (21.1%) relative to the total number of physicians. The proportion of male PSs has continued to be relatively high, suggesting a persistent male-dominant tendency [1] in this area as well.

### Policy suggestions for the recruitment of physician scientists

A decrease in the number of younger PSs is a global issue. Recent proposals to address this issue in the U.S. [26, 27] suggest the following: the period required for students to become independent researchers is shortened; diversity in training programs are guaranteed; the number of students enrolled in diversity in training programs is increased; academia takes the lead in setting up PS career development offices, in order to secure PSs and provide career support to PS candidates; and a new system for procuring funding from the industry is developed.

In Japan, since the proposal statements by the Association of Japanese Medical Colleges [8] and academic societies for basic medicine mentioned above were issued [9], from 2011 onwards MEXT developed MD-PhD courses and postgraduate PS training courses in medical schools across Japan, intending to facilitate PS training [14]. The Plan for Facilitating Medical Research and Development announced in 2014 states that in order to make continuous progress in medical research and development, it is necessary to strengthen basic research so that innovation is constantly generated [28]. In line with this, MEXT increased the number of enrollments in medical schools that have set up MD-PhD courses and agreed to provide financial support to these educational organizations. The Ministry also set up the Program for Nurturing Physicians in Basic Research, to build a collaboration system between medical colleges for nurturing physicians specializing in basic research. The program also has the aims of facilitating human resources exchanges both within and outside Japan and building career paths for physicians engaging in basic research (employment in international organizations and research institutions, and securing tenure positions). However, a budget of only 1 million dollars was allocated to the Program in 2017, and from among 80 medical schools in Japan, 39 have set up MD-PhD courses. Considering the current shortage of younger PSs, these efforts may have not yet generated sufficient achievements.

The salaries of PSs in Japan are lower than those of physicians working in health care organizations [29]. Moreover, it has been pointed out that certain universities illegally require physicians undertaking PhD courses to provide clinical practice without compensating them for these activities [30]. A low salary for young PSs can be a major cause of PS shortages.

More research is required regarding the reasons for the different career choices that physicians make, and further action is required to keep PSs from leaving their careers. A system that facilitates the smooth transition between clinical practice and medical research could help maintain PS human resources.

### Limitation and strength

This study has several limitations. First, the workplace was self-reported; subsequently, misclassification may have occurred. Second, I could not acquire data to distinguish part-time PSs. Third, this study did not differentiate staff from postgraduate students due to the limitations of the questionnaire. Fourth, this study was only concerned with association and could not ascertain causality. The use of interviews and questionnaires could facilitate more comprehensive research. Fifth, in this study, SMAs were classified into three categories based on population and population density. Thus, the results might change if the classification method were to change. Sixth, because the study is based on secondary use of national survey data, it is too less variables in the regression models might affect the robustness of the model.

The strength of the present study is that it used the individual data of the national census; therefore, the sample size was large and the capture rate was high. While there is insufficient information on the distribution and retention trends of PSs, the present study provided information that may contribute to investigations into the circumstances of PSs and measures for securing these physicians in the future.

## Conclusion

This study demonstrated the following: the number of PSs has only slightly increased, compared to the number of overall physicians; if the decreasing tendency in the number of younger PSs is not addressed, the total number of PSs will drastically decrease in the future. The Japanese government has implemented various measures to address the decrease in younger PSs, such as setting up MD-PhD courses, but these measures have not been sufficiently effective.

In order to secure a stable number of PSs, it is important to consider various measures that take into account similar discussions and policies in the U.S. and other countries. The possible measures include increasing opportunities for medical students and younger physicians to understand the role of PSs and the benefit of working as a PS, providing career paths for PSs and securing PS positions, and increasing compensation for PSs.

### Policy suggestions

Subsequently, based on the results of the present study, what follows provides recommendations for increasing and retaining the number of PSs from three perspectives.

First, it is important to increase the exposure of younger physicians to PS as a career as well as the benefits of working as a PS by increasing the number of universities that have MD-PhD courses and the number of enrollments in these courses. Providing undergraduate and postgraduate research practice sessions and internship programs can also increase this exposure. Additionally, it may be worth setting up an office in each university with a specific focus on PS as a career for younger physicians.

Second, it is necessary to consider measures, such as improving PS positions, extending employment contracts for PSs, and shortening training periods, by understanding PSs’ situations and requirements in detail. The issue of securing various career paths for PSs is also worth considering; this would include developing human resources networking as well as cross-appointment systems with research institutions and private companies both within and outside Japan, and re-employing retired PSs.

Third, PS compensation should be reviewed and, if necessary, increased. It might be effective to improve scholarship programs for undergraduate and postgraduate medical students. It might also be effective to use national research funding for human resources expenses, although doing so is not currently permitted by the government. Additionally, increasing operational efficiency by shifting administrative tasks from researchers to non-researchers so that researchers can spend less time on non-research-related work is recommended.

## Data Availability

Not applicable.

## Abbreviations

MEXT: Minister of Education, Culture, Sports, Science and Technology
MHLW: Ministry of Health, Labour and Welfare
NIH: National Institute of Health
PSs: Physician-scientists
SMAs: Secondary Medical Areas
